# Pennsylvania State Medical Society

**Published:** 1850-05

**Authors:** 


PENNSYLVANIA STATE MEDICAL SOCIETY.

*Philadelphia*, April 17th, 1850.

The Society met in the Hall of the Controllers of the Public Schools, in the Athenæum building, Sixth Street, in the city of Philadelphia, pursuant to adjournment, and was called to order by Dr. Wilmer Worthington, Vice President, at 11½ o'clock, A. M.

At twelve o'clock, Professor Jackson addressed the Society in a speech marked with great power of thought, apt illustration, and felicity of expression. He stated, in substance, that it was two years since the Society was founded in the city of Lancaster, and expressed the confident belief that important results would flow from the organization of the medical profession of the State. The arbitrary governments of Europe have regulated the medical profession, and by regal statutes established medical colleges; but they are of a character incompatible with our free institutions. We do not want Legislative or Executive interference, as the medical profession alone are capable of legislating understandingly, in those things which affect their usefulness, standing and interests. Allusion was made to the evils resulting from empirics assuming the name and honors of the Doctorate, and their pretended claims silently recognized by an unreflecting public. A great want in medicine is a large collection of trustworthy facts, embracing many thousand cases, and extended over a considerable period of time. Isolated cases prove nothing. They are empirical. Laws are required, and laws can only be discovered from accumulated thousands of individual facts. In regard to the preparatory studies of medical students, he thought that a knowledge of French and German was of more importance than a knowledge of Greek and Latin. After showing, at length, the elevation which should be attained by the medical profession, and the means to be employed in attaining it, he remarked, in conclusion, that medicine will then have accomplished its important mission in this world, corresponding to a civilization far different from the present. This is the great work charged on us. It is now being organized; its scattered elements of power are concentrating—combined exertion and united effort must hasten its progress. In this work, we all have an allotted though small task to perform. Let the word then be, "forward."

On motion of Dr. Kerfoot, of Lancaster, the thanks of the Society were tendered to Professor Jackson, for his able and eloquent address, accompanied with a request that he furnish a copy of the same for publication.

A motion was made that delegates be received from the Medico-Chirurgical Society of Philadelphia, but after lengthy debate the motion was finally withdrawn.

The roll, being submitted by the committee, was called, and 58 delegates answered to their names.

On motion, the Medical officers of the Army and Navy, and medical gentlemen from other States and counties, and associate members, were invited to seats.

Dr. Jackson presented a series of resolutions relative to the assimilated rank of medical officers in the Navy, which was granted in 1846 by order of the Secretary of the Navy. In the army it is granted by a law of Congress. Many officers of the line of the Navy are now attempting to degrade medical officers by depriving them of that assimilated rank. The following resolutions were adopted and ordered to be forwarded to the War and Navy departments, and to the Chairman of the War and Navy Committees in both houses of Congress.

*Resolved*, That the State Medical Society of Pennsylvania cannot regard with indifference the condition and position assigned to members, of the profession, who are included in the military organizations of the country; and therefore regards, with satisfaction, the law which confirms the assimilated rank of Medical officers in the Army, previously conferred by the regulations of the War Department.

*Resolved*, That the members of the State Medical Society of Pennsylvania approve of the general order issued by the Secretary of the Navy, August 31st, 1846, which assigns to Medical officers in the Navy an assimilated rank; and that their influence will be used to sustain their naval brethren in a position alike due to them and the profession of which they are members.

*Resolved*, That inasmuch as the Medical officers of the Army have been assigned an assimilated rank by law, it is right and proper, that Medical officers of the Navy should also be assigned a definite rank by act of Congress, and this body, therefore, respectfully and earnestly invites the attention of the Senate and House of Representatives of the United States to the subject.

*Resolved*, That a copy of these resolutions be forwarded to the Secretaries of War, and of the Navy, through the chiefs of the Medical Department, of each service, and to the chairman of the military and naval committees in each House in Congress.

At two o'clock, P. M. on motion, the Society adjour ned to mee a four o'clock, P. M.

The subjoined, resolution, offered by Dr. Isaac Parrish, was passed:

*Resolved*, That the attention of the County Medical Societies throughout the State, be called to the importance of furnishing reports on the state of Health, the Medical Topography, the occurrence and progress of contagious and epidemic diseases within their respective limits, and such other medical intelligence as may be of importance to the State Society.

On motion of Dr. Henry Gibbons, it was

*Resolved*, That a Standing Committee be appointed to receive the the annual reports from the County Societies, and present them to the State Society, accompanied with such remarks as they may deem interesting.

Five being resolved upon as the number of the committee, the Chair appointed the following gentlemen to constitute the same—Drs. I. Parrish, J. L. Atlee, A. Stillé, H. S. Patterson, and Worthington.

Dr. Atlee, of Lancaster, then announced in appropriate terms the demise of Dr. C. H. Matthews, of Bucks county, one of the Vice Presidents of the Society, and after paying a brief tribute to his worth, offered a series of resolutions expressive of the feelings of the Society upon the occasion. They were adopted unanimously.

Dr. Hays, the Corresponding Secretary, presented a report from the Erie County Medical Society, detailing the organization and proceedings of that body. The document, which was a long and interesting one, was read and ordered to be entered upon the Society's archives.

Dr. Stillé offered the following resolution which was adopted:

*Resolved*, That the County Societies be requested, at the earliest day, to procure an enumeration of the regular medical practitioners within their limits, distinguishing between those who are graduates of medical schools and those who practice medicine but who are not graduates, and to state also, the number of irregular practitioners, distinguishing between the adherents of the several false systems which prevail.

Dr. Stillé also offered the following resolution, which was lost—ayes 20, nays 22.

*Resolved*, That the censors of the several districts be requested to correspond with physicians in the counties where no medical Societies exist, soliciting such information as they possess in reference to the subjects of the resolutions above.

The reason for the rejection of this resolution were, that the information thus gathered would be merely opinionative, and might do injury to individuals.

Dr. Hays offered the following:

*Resolved*, That it be recommended to the several County Societies to adopt some regulations by which members to whom application is made by a person desirous of becoming a student of medicine, should be satisfied that the applicant has received a good English education, possesses respectable attainments in the Latin language, and sustains a good moral character, and that it is recommended to the County Societies to make it the duty of a member having students in his office, from time to time to make such examinations of their progress as shall enable him to give them conscientious certificates of their abilities when they leave the office for the lecture-room.

After some discussion this resolution was laid on the table.

The following resolution was substituted for the above, and adopted:

*Resolved*, That it be recommended that members satisfy themselves, either by personal inquiry or written certificate of competent persons, before receiving young men into their offices as students, that they are of a good moral character, and that they have acquired a good English education, a knowledge of natural philosophy and the elementary mathematical sciences, including geometry and algebra; and such an acquaintance, at least, with the Latin and Greek languages as will enable them to appreciate the technical language of medicine, and read and write prescriptions; and that it is recommended to the County Societies to make it the duty of a member having students in his office, from time to time to make such examinations of their progress as shall enable him to give them conscientious certificates of their abilities when they leave the office for the lecture room.

Upon these resolutions a very interesting discussion arose as to the utility of requiring a proficiency in algebra, etc.

Dr. Worthington offered the following, which, after some discussion, was adopted:

Whereas, it is asserted and confidently believed by a portion of the public, that it is the practice of some physicians and apothecaries to enter into a collusive agreement, by which the former are to receive a per centage upon all prescriptions sent to the latter, and in this way bring dishonor upon the Medical profession: Therefore,

*Resolved*, That this Society regards all collusion between physicians and apothecaries, whether with a view to pecuniary profit or patronage, as opposed to every principle of that moral code which the profession have adopted for their government, and that no physician known to be guilty of such collusion should be entitled to the confidence and professional intercourse of medical men.

*Resolved*, That the delegates representing this Society in the American Medical Association, be requested to bring this subject before that body at its next annual meeting, and endeavor to procure such alteration in our code of ethics as will exclude all physicians guilty of such practice from medical consultations.

During the debate upon these resolutions, it was stated that there are in the city and county 380 physicians.

Philadelphia was agreed upon as the next place of meeting.

On motion of Dr. Condie, the article of the constitution respecting the time of meeting was altered to the month of May, instead of April. On motion of the same gentleman, the next annual session was fixed upon the last Wednesday of May, instead of April.

On motion of Dr. Green, the section of the constitution disqualifying from membership physicians guilty of certain medical offences, was amended so as to include in it the words "or who shall enter into a collusive agreement with an apothecary to receive compensation or patronage for sending prescriptions to said apothecary."

Dr. Patterson, from the Committee appointed at the last session to draft an address to the profession throughout the State, reported that the duty had been performed, and the address printed with the proceedings of last session.

Dr. West, from the committee on a Medical Beneficiary Fund and mutual assurance among Physicians, read a written report of considerable length, to which was appended several resolutions approving of the spirit of medical beneficial Societies and mutual assurance among physicians, but declaring that those objects could be more effectually attained by the various local and county Societies, to whom the subject was therefore referred, with an earnest recommendation from this Society that they take immediate action, and report the result at the next annual session. The report was approved, directed to be entered on the minutes, and the resolutions adopted.

Dr. Worthington, from the Committee on the Registration of births, marriages, and deaths, reported an act for that purpose, which it was proposed to lay before the Legislature, with a recommendation that it be enacted into a law.

This bill makes it the duty of the Assessors of the several Wards and Townships throughout the State to ascertain the number of births, marriages, and deaths in their jurisdiction every year, which are to be returned to the Register of Wills in each county. The act also provides a penalty, in order to secure the faithful performance of this duty, and makes the record of these births, marriages and deaths evidence in courts of justice.

On motion, the committee was continued, and instructed to urge upon the Legislature the passage of the bill.

Prof. Samuel Jackson, from the Committee on Small Pox and Varioloid, made a lengthy report of the facts ascertained by that Committee relative to the prevalence of those diseases. The Professor read the statistics of the number of deaths from year to year in Philadelphia of these diseases, and compared them with the statistics of London, both of which went to show that the epidemic obeys certain laws appearing, increasing, and diminishing at intervals of from three to five years.

The statistics of the disease in this city we give below. In the years 1804, 5, 6, there were no cases of death. In 1807 there were 32; in 1808, 145; 1809, 101; 1810, 33; 1811, 117; in 1812, 13, 14, 15, no deaths; in 1816, 97; in 1817, 52; 1818, 8; 1819, 1; 1820, 21; 1822, no cases; 1823, 160; 1824, 325; 1825, 6; 1826, 3; 1827, 100; 1828, 107; 1829, 81; 1830, 86; 1831, 18; 1832, 43; 1833, 168; 1834, 212; 1835, 106; 1836, 86; 1837, 81: 1838, 45; 1839, 5; 1840, 63; 1841, 259; 1842, 156; 1843, 36; 1844, 17; 1845, 190; 1846, 251; 1847, 9; 1848, 100. The report was received, and ordered to be published, and the committee continued with power to fill vacancies.

The Committee on nominations reported the following nominations for officers of the Society for the ensuing year:—President, Dr. Wilmer Worthington, of Chester county; Vice Presidents, Drs. Allen, of Bucks; Gries, of Berks; Innes, of Northampton; Ludens, of Huntingdon. Recording Secretaries, Drs. Ehler, of Lancaster, and Patterson, of Philadelphia. Corresponding Secretary, Dr. Hays, of Philadelphia. Treasurer, Dr. Fox, of Philadelphia. Delegates to the American Medical Association for 1850—Drs. Norris and Yardley, of Philadelphia; Burrows, of Lancaster; Wood, of Philadelphia; Betton, of Philadelphia; and Ely of Bucks. Delegates for 1851—Drs. S. Jackson, Philadelphia; Kerfoot, Lancaster; Abernethy, Northampton; Worthington, Chester; McCullough, Huntingdon: Nagel, Berks. Censors the same as last year.

On motion of Dr. Kerfoot, the President elect was requested to deliver an address at the opening of the next annual session of the Society.

Dr. Carpenter offered the subjoined resolution:

Whereas, one of the chief objects in the formation of the State Medical Society, was to effect a full and complete organization of the profession within its limits, whereby its interests and usefulness would be more fully extended amongst its members and the public generally, it is consequently of much importance to effectually arouse the attention of the profession in counties yet unorganized, so as to induce them to take such action as will early effect the desired object.

Therefore, *Resolved*, That the Corresponding Secretary be requested to ascertain the names of one or more physicians in such counties, and send them a copy of the proceedings of this meeting, with an urgent request to take the necessary steps to insure their representation at the next meeting of the Society.

On motion of Dr. Hays, the Recording Secretaries were directed to publish the proceedings of this session, with the Constitution of the Society, the address of Professor Jackson, and the address to the profession published by the Committee.

The following resolution was offered by Dr. Carpenter, of Lancaster, and adopted :

*Resolved*, That the thanks of the State Medical Society be presented to the delegation of Philadelphia for the comfortable arrangements provided for its meeting, as well as the hospitality and kindness extended to its members.

Dr. Ehler offered the following, which was adopted :—

*Resolved*, That a vote of thanks be tendered to the late officers of the State Medical Society for the gentlemanly and efficient manner in which they have fulfilled the duties of their respective offices.

The President, Professor Jackson, returned his acknowledgments, on his own behalf and that of the officers, for the compliment, in a few very happy remarks. He then inducted into office the President elect, Dr. Wilmer Worthington, of Chester county, who, in a short speech, returned thanks for the honor conferred upon him.

The Society adjourned *sine die*.

